# Is endocan correlated to ARDS severity or an epiphenomenon of thrombo-embolic disease in COVID

**DOI:** 10.1186/s13054-021-03850-9

**Published:** 2021-12-14

**Authors:** Patrick M. Honore, Sebastien Redant, Thierry Preseau, Bogdan Vasile Cismas, Keitiane Kaefer, Leonel Barreto Gutierrez, Sami Anane, Rachid Attou, Andrea Gallerani, David De Bels

**Affiliations:** 1 ICU Department, Centre Hospitalier Universitaire Brugmann, Brussels, Belgium; 2 ED Department, Centre Hospitalier Universitaire Brugmann, Brussels, Belgium; 3 ICU Department, Centre Hospitalier Universitaire Brugmann-Brugmann University Hospital, Faculty of Medicine,, ULB University, Place Van Gehuchtenplein, 4, 1020 Brussels, Belgium

**Keywords:** Endocan, ARDS severity, Pulmonary embolism

With great interest, we read the recently published paper by Pascreau et al. concluding that a high blood endocan profile during COVID‑19 distinguishes moderate from severe acute respiratory distress syndrome (ARDS) [1]. In a recent study including 46 patients with a diagnosis of pulmonary thromboembolism (PTE) and a control group of 25 healthy individuals [2], there was a significant difference in the serum endocan levels between the patients and the control group (321.93 ng/l and 192.77 ng/l, respectively; *p* < 0.030) [2]. Endocan is likely a good marker for PTE, a frequent phenomenon in COVID-19 [3]. In a prospective postmortem evaluation of 735 consecutive SARS-CoV-2-associated deaths, on autopsy (*n* = 283) it was found that the majority died of pneumonia and/or diffuse alveolar damage (73.6%) but thromboses were found in 39.2% and PTE in 22.1% [3]. Another study demonstrated significant associations between PTE and not only mechanical ventilation (OR = 3.71, 95% CI 2.57–5.36), but also intensive care unit admission (OR = 2.99, 95% CI 2.11–4.23), circulating D-dimer [mean difference (MD) = 5.04 µg/mL, 95% CI 3.67–6.42) and c-reactive protein (CRP) (MD = 1.97 mg/dL, 95% CI 0.58–3.35) [4]. Those characteristics are similar to those found in the Pascreau study (high CRP, high D-dimers and mechanical ventilation in ARDS COVID patients) [1]. As there is no indication they excluded PTE in their patient cohort, it seems plausible that Pascreau et al. are describing the consequences of thromboses and PTE in ARDS COVID patients [1]. The explanation for endocan levels is therefore more likely to be found here than as a marker of ARDS severity.

## Data Availability

Not applicable.

## Abbreviations

ARDS: Acute respiratory distress syndrome
PTE: Pulmonary thromboembolism
